# The Wnt Co-Receptor Lrp6 Is Required for Normal Mouse Mammary Gland Development

**DOI:** 10.1371/journal.pone.0005813

**Published:** 2009-06-05

**Authors:** Charlotta Lindvall, Cassandra R. Zylstra, Nicole Evans, Richard A. West, Karl Dykema, Kyle A. Furge, Bart O. Williams

**Affiliations:** 1 Laboratory of Cell Signaling and Carcinogenesis, Van Andel Research Institute, Grand Rapids, Michigan, United States of America; 2 Flow Cytometry, Van Andel Research Institute, Grand Rapids, Michigan, United States of America; 3 Computational Biology, Van Andel Research Institute, Grand Rapids, Michigan, United States of America; Roswell Park Cancer Institute, United States of America.

## Abstract

Canonical Wnt signals are transduced through a Frizzled receptor and either the LRP5 or LRP6 co-receptor; such signals play central roles during development and in disease. We have previously shown that Lrp5 is required for ductal stem cell activity and that loss of *Lrp5* delays normal mammary development and Wnt1-induced tumorigenesis. Here we show that canonical Wnt signals through the Lrp6 co-receptor are also required for normal mouse mammary gland development. Loss of *Lrp6* compromises Wnt/β-catenin signaling and interferes with mammary placode, fat pad, and branching development during embryogenesis. Heterozygosity for an inactivating mutation in *Lrp6* is associated with a reduced number of terminal end buds and branches during postnatal development. While *Lrp6* is expressed in both the basal and luminal mammary epithelium during embryogenesis, *Lrp6* expression later becomes restricted to cells residing in the basal epithelial layer. Interestingly, these cells also express mammary stem cell markers. In humans, increased *Lrp6* expression is associated with basal-like breast cancer. Taken together, our results suggest both overlapping and specific functions for Lrp5 and Lrp6 in the mammary gland.

## Introduction

Wnt signaling plays key roles in embryogenesis and in adult tissue homeostasis of metazoan animals [1]. The extracellular Wnt signal stimulates numerous intracellular signal transduction cascades, including the canonical Wnt/β-catenin pathway, which regulates gene expression in the nucleus, and a number of noncanonical pathways, which regulate many other aspects of cell biology including cell migration, adhesion, and polarity [2]–[4]. Mutations of the genes involved in Wnt signaling cause congenital defects in humans, and inappropriate activation of the Wnt/β-catenin pathway has been linked to the development of human cancer [5]–[7]. An increasing number of studies have shown that Wnt/β-catenin signaling regulates the self-renewal and differentiation of adult stem cells, raising the possibility that this process is subverted in cancer [8]–[10].

Activation of the Wnt/β-catenin pathway is initiated by the binding of Wnt proteins to cell surface receptors composed of a member of the Frizzled (Fzd) protein family and one of the two low-density lipoprotein receptor–related proteins, LRP5 or LRP6 (reviewed in [11]). Signaling from Wnt receptors leads to inactivation of a cytoplasmic protein complex that normally catalyzes the phosphorylation and subsequent destruction of β-catenin. Canonical Wnt signaling thus induces stabilization of cytosolic β-catenin. A fraction of β-catenin then enters the nucleus, binds to transcription factors such as those of the LEF-1/TCF family, and modulates the transcription of specific target genes (see The Wnt Homepage at http://www.stanford.edu/~rnusse/wntwindow.html). 

The initiation of canonical Wnt/β-catenin signaling requires LRP5 or LRP6 [12]–[14]; in contrast, noncanonical Wnt pathways are usually independent of these two proteins [3], [4]. LRP5 and LRP6 are highly homologous and exhibit functional redundancy both *in vitro* and *in vivo* [14]–[17]. However, loss-of-function studies in animals show that Lrp5 and Lrp6 also have unique roles for which the other cannot compensate. In mice, disruption of *Lrp6* causes severe developmental defects [13]. The defects reflect a composite of some of the Wnt mutant phenotypes and include a deletion of caudal midbrain, axis truncation, and limb patterning defects. Neonatal lethality prevents a thorough analysis of the consequences of *Lrp6* deficiency in the adult mouse. Disruption of *Lrp5* does not cause gross developmental abnormalities, but abnormalities have been identified in a number of different tissues [17]–[20]. We have previously shown that Lrp5 is required for ductal stem cell activity, which is apparent by the failure of *Lrp5^−/−^* mammary epithelial cells to colonize in transplantation experiments [21]. Consequently, loss of *Lrp5* is associated with delayed mammary gland development and Wnt1-induced tumorigenesis [21]. Here we show that canonical Wnt signals through the Lrp6 co-receptor are also required for normal mammary gland development.

## Results

### *Lrp6* expression in the mammary gland

The targeting vector used to create the *Lrp6^−/−^* mouse strain contained the β*-galactosidase* (β*-gal*) gene. As a result, β*-gal* expression is directed from the *Lrp6* promoter in *Lrp6^+/−^* and *Lrp6^−/−^* mice and can be used as a surrogate marker for *Lrp6* expression [13]. Several substrates are available to detect β*-gal*, including 5-bromo-4-chloro-3-indolyl-β-D-galactopyranoside (X-gal) and 9*H*-(1,3-dichloro-9,9-dimethylacridin-2-one-7-yl)-β-D-galactopyranoside (DDAOG).

**Figure 1:**
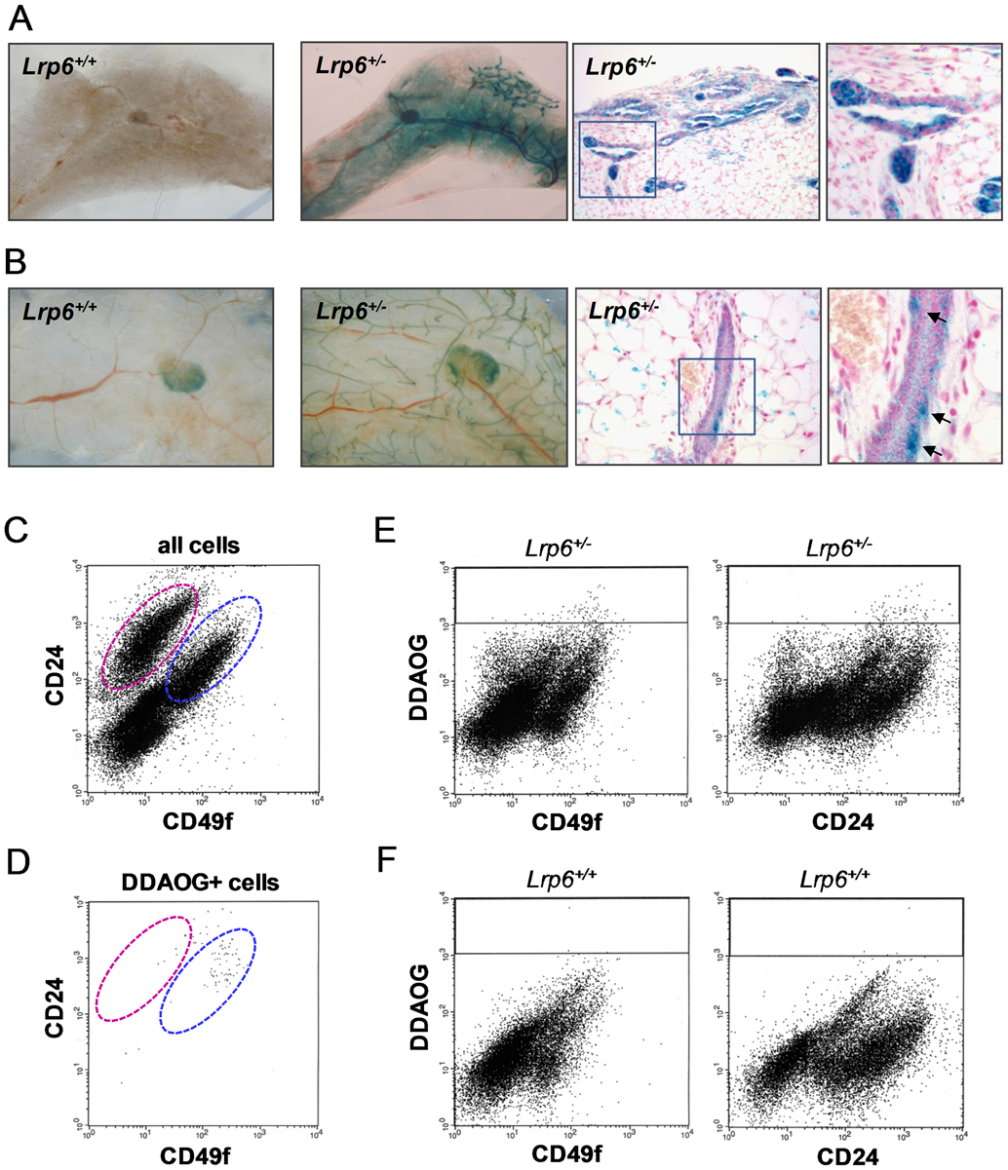
Expression pattern of *Lrp6* in the mammary gland. β*-gal* expression directed from the *Lrp6* promoter in *Lrp6^+/−^* mice was used as a surrogate marker for *Lrp6* expression. We used the β*-gal* substrates X-gal and DDAOG to identify cells that express *Lrp6*. Shown in (A-B) are X-gal-stained mammary whole mounts and 5-µm sections from 2-day-old (A) and 12-week-old (B) female mice. (A) *Lrp6* promoter-driven β*-gal* expression is visible as blue staining in both the basal and luminal mammary epithelium and in the mammary fat pad of newborn mice. (B) In older mice, β*-gal*-expressing cells are primarily identified within the basal epithelial cell layer and in the mammary fat pad. The *arrows* indicate typical cells that stained blue with X-gal. No staining was detected in *Lrp6^+/+^* mammary glands, which were used as negative controls for X-gal staining. (C-F) Representative FACS results of DDAOG-stained and CD24/CD49f antibody–labeled mammary epithelial cells. The β*-gal*-cleaved product of DDAOG has far-red fluorescence and was used to detect cells with *Lrp6* promoter–driven β*-gal* expression. (C) The luminal and basal cell compartments are marked by pink and blue dashed lines, respectively. (D) 80% of the DDAOG-positive cells are found within the basal epithelial cell compartment. (E-F) DDAOG gating strategy. The DDAOG gate is indicated by the black line. The *Lrp6^+/−^* sample (E) has 0.33% DDAOG-positive cells; the *Lrp6^+/+^* negative control sample (F) has 0.01% DDAOG-positive cells.

In order to determine the expression pattern of *Lrp6* in the mammary gland, we first stained mammary whole mounts from *Lrp6^+/−^* and *Lrp6* ^+/+^ females of different ages (newborn, juvenile 5-week, adult 12-week, and days 12.5 and 18.5 of pregnancy) with X-gal. β*-gal* expression was detected in the *Lrp6^+/−^* mammary epithelium, stroma, and fat pad at all analyzed time points (Fig. 1A–B and data not shown). However, the expression pattern differed between mammary glands collected from newborn females versus glands collected from juvenile, adult, and pregnant females. The mammary epithelium contains a basal cell layer of mostly myoepithelial cells and a luminal cell layer of keratin 8/18–positive epithelial cells. During embryogenesis and until a few days after birth, β*-gal* expression was detected in both the basal and luminal mammary epithelium (Fig. 1A). In juvenile, adult, and pregnant females, however, β*-gal* expression was primarily seen in cells residing within the basal epithelial cell layer of mammary glands (Fig. 1B). Abundant *Lrp6* expression was seen in the mammary fat pad at all developmental time points analyzed (Fig. 1A–B and data not shown). *Lrp6^+/+^* mammary whole mounts were used as a negative control for the X-gal staining (Fig. 1A–B).

To further characterize the expression pattern of *Lrp6* in the adult mammary epithelium, we performed fluorescence-activated cell sorting (FACS) analysis on single-cell suspensions isolated from mammary glands of 3-month-old *Lrp6^+/−^* and *Lrp6* ^+/+^ females. Because hematopoietic and stromal cells can make up at least 50% of such cell suspensions, we first removed cells expressing cell surface antigens of hematopoietic and endothelial origin (CD45, Ter119, and CD31). The epithelial cell–enriched fractions were then labeled with the CD24 (heat-stable antigen) and CD49f (α6 integrin) cell surface antigens. We used the fluorescent β*-gal* substrate DDAOG [22] to detect cells that express *Lrp6*. The basal and luminal epithelial subpopulations, as well as a cell fraction enriched for mammary stem cells, can be visualized by the relative expression of CD24 and CD49f [23], [24] (Fig. 1C). In concordance with the X-gal staining, we found by FACS that the majority of DDAOG-positive cells resided in the basal subpopulation (Fig. 1D). These DDAOG-positive cells expressed the highest levels of CD49f and moderate levels of CD24 (Fig. 1C–D). This observation was of particular interest because mammary epithelial cells with stem cell properties typically exhibit this expression pattern [23]. *Lrp6^+/+^* mammary epithelial cells were used as a negative control for the DDAOG staining (Fig. 1F).

### Impaired mammary development in *Lrp6^−/−^* embryos

**Figure 2:**
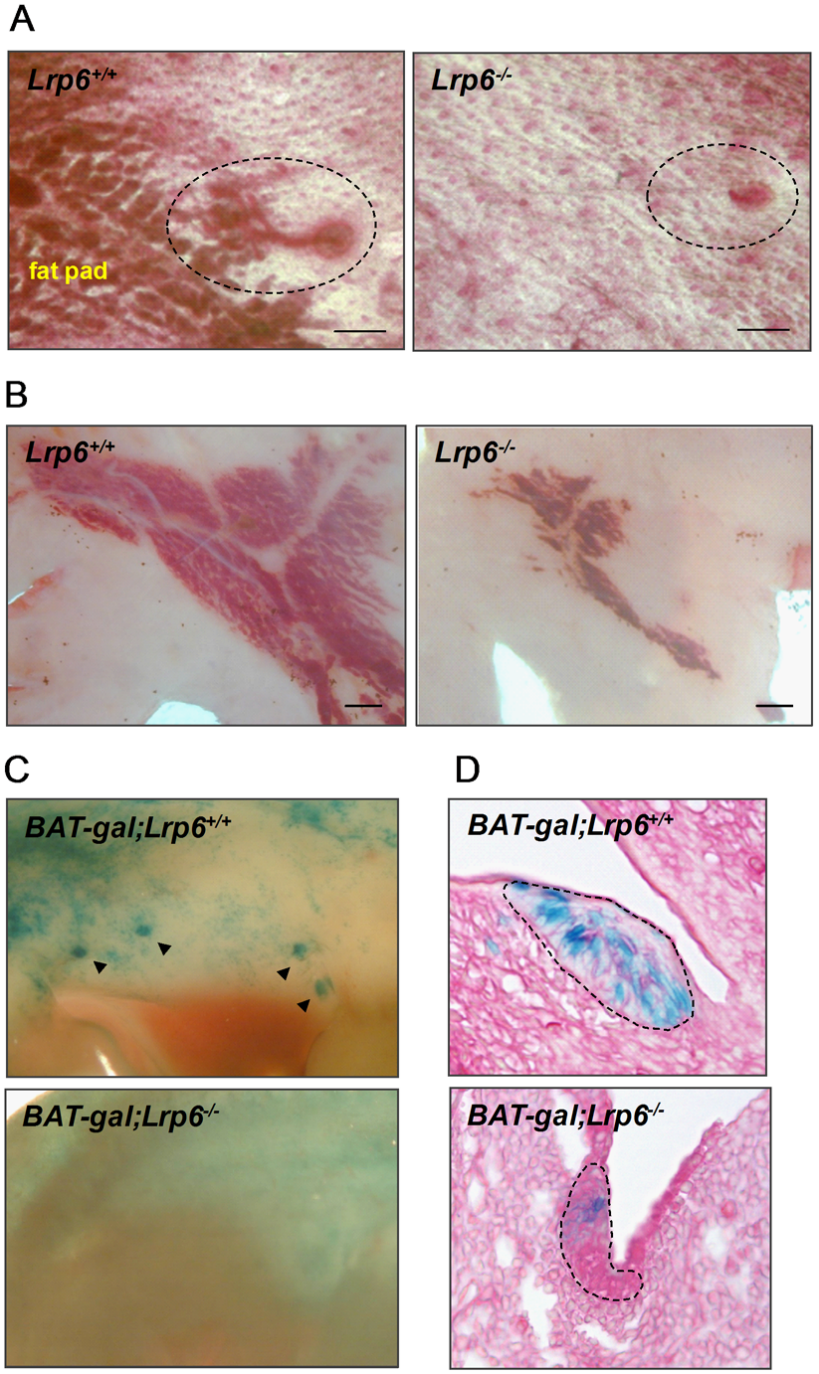
*Lrp6* is required for embryonic mammary development. (A) Carmine-stained skin pads of inguinal mammary glands at E18.5. While in the *Lrp6^+/+^* mammary gland the nipple, rudimentary ductal tree, and fat pad are all normally developed, the *Lrp6^−/−^* mammary gland contains a small nipple, a single ductal out-growth, and an abnormally small fat pad. *Dashed lines* indicate inguinal epithelium. (B) Oil red O staining of mammary fat pads from *Lrp6^+/+^* and *Lrp6^−/−^* embryos. The *Lrp6^−/−^* fat pad is abnormally small compared to that of the littermate control. *Scale bar*: 0.5 mm. (C-D) X-gal-stained *BAT-gal* embryo whole mounts (C) and histology sections of mammary placode (D) at E12.5. Cells expressing BAT-gal are stained blue. (C) X-gal stains the mammary placodes of *BAT-gal;Lrp6^+/+^* embryos dark blue. *Arrow heads* indicate mammary placodes number 2, 3, 4, and 5. Mammary placodes are not readily visible on X-gal-stained *BAT-gal;Lrp6^−/−^* embryo whole mounts. (D) On the histological level, the mammary placodes of *BAT-gal;Lrp6^−/−^* embryos are significantly smaller and exhibit fewer cells with *BAT-gal* expression than the mammary placodes of littermate controls. *Dashed lines* indicate inguinal placodes.

*Lrp6^−/−^* mice die shortly after birth. To determine the effect of *Lrp6* deficiency on the rudimentary mammary gland that develops by birth, we collected ventral skin from *Lrp6^−/−^* (*n* = 5) and *Lrp6^+/+^* (*n* = 10) embryos at E18.5. Normally at this stage, the mammary gland is composed of a ductal tree consisting of a primary duct with secondary and tertiary branches surrounded by a fat pad. We found that the while all 10 mammary glands were present in *Lrp6^−/−^* embryos, they were underdeveloped. The nipples were considerably smaller than those of littermate controls and the mammary epithelium typically consisted of a single duct (Fig. 2A). In a minority of inguinal *Lrp6^−/−^* mammary glands, the epithelium formed two short branches at the extremity of the main duct (not shown). During dissection and carmine staining we noticed that the adipose tissue forming the fat pad was abnormally small and underdeveloped in *Lrp6^−/−^* embryos (Fig. 2B). In contrast, all *Lrp6^+/+^* mammary glands showed secondary and tertiary branches surrounded by a fat pad (Fig. 2A–B).

The ductal tree at E18.5 evolves from the epithelial mammary placode, which has formed by E12.5. To determine whether Lrp6-mediated Wnt signaling is required for the development of mammary placodes, we analyzed *Lrp6^−/−^* and *Lrp6^+/+^* littermate whole embryos at E12.5. To assess the activity of Wnt/β-catenin signaling pathway in this context, we intercrossed *Lrp6^+/−^* mice to *BAT-gal* transgenic mice. *BAT-gal* mice carry a reporter gene which contains a β*-gal* gene under the transcriptional control of LEF/TCF sites [25]. It is important to note that β*-gal* expression induced by *BAT-gal* reporter gene activity can be discriminated from β*-gal* expression induced by *Lrp6* promoter activity due to a large difference in the time it takes to detect the β-gal by X-gal staining. While *BAT-gal*-associated β*-gal* expression is detected within 20 minutes of X-gal staining, *Lrp6*-associated β*-gal* expression is not detected until after several hours of X-gal staining. Our results showed that loss of *Lrp6* compromised Wnt/β-catenin signaling in the developing mammary placodes and interfered with their formation. *BAT-gal* reporter gene activity was significantly reduced in *Lrp6^−/−^* embryos relative to littermate controls (Fig. 2C). On the histological level, the mammary placodes of *Lrp6^−/−^* embryos were significantly smaller and contained fewer cells with reporter gene activity (Fig. 2D).

### Postnatal mammary development in *Lrp6^+/−^* females

**Figure 3:**
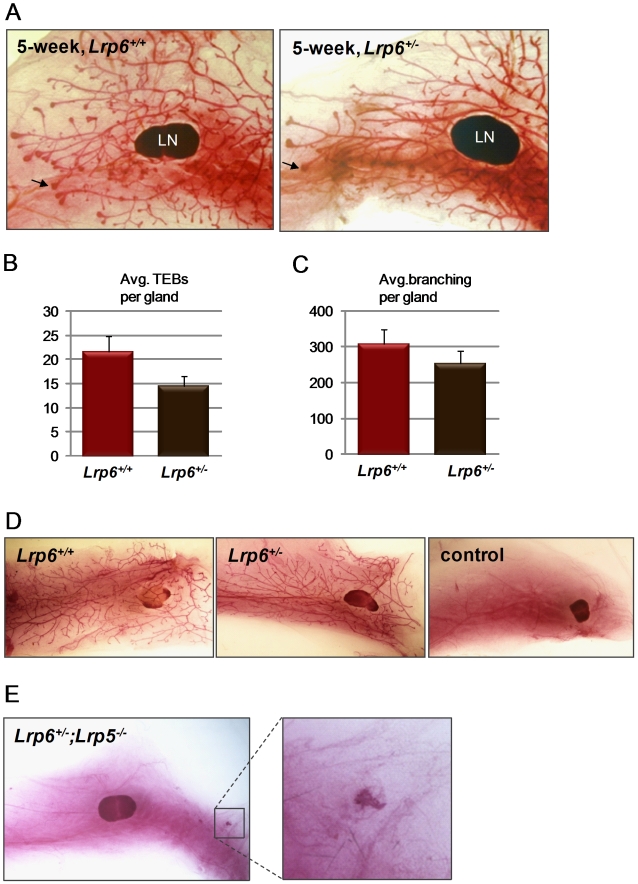
Haploinsufficiency for *Lrp6* in postnatal mammary development. (A) Representative mammary whole-mount preparations are shown for juvenile (5-week-old) mice. The *arrows* indicate typical terminal end buds; *LN*, lymph node. (B) The result of morphometric analysis of the average number of TEBs at 5 weeks and (C) of branches per gland at 11 weeks. At least 10 animals of each genotype were analyzed for each time point. In the absence of one copy of *Lrp6*, the number of TEBs is reduced by 33% (*p* = 1.3×10^−6^, 2-tailed *t* test assuming unequal variances), and the number of branches per gland is reduced by 17% (*p* = 8.4×10^−3^, 2-tailed *t* test assuming unequal variances) compared with *Lrp6* ^+/+^ littermate controls. (D) Mammary whole mounts containing ductal colonization originating from transplanted *Lrp6^+/+^* or *Lrp6^+/−^* mammary epithelial cells. Also shown is a transplantation control whose inguinal fat pads were cleared of endogenous epithelium but not injected with mammary cells. (E) The inguinal mammary gland from an adult *Lrp6^+/−^;Lrp5^−/−^* female. The mammary gland contains a fat pad and a nipple with associated epithelium but lacks a ductal tree. *Box* indicates the nipple epithelium.

To determine if the loss of one allele affects postnatal mammary development, we collected and examined inguinal mammary whole mounts from juvenile (5-week) and adult (11-week) *Lrp6^+/−^* and *Lrp6^+/+^* female littermates. No differences in ductal extension were found at 5 weeks (Fig. 3A). However, the number of terminal end buds (TEBs) was significantly reduced in *Lrp6^+/−^* mammary glands (Fig. 3A–B). TEBs are club-shaped epithelial thickenings at the distal ends of growing ducts and are the sites of the most rapid cell proliferation. We found that the number of TEBs was reduced by 33% in juvenile *Lrp6^+/−^* mice compared with littermate wild-type mice (*p* = 1.3×10^−6^). The branching complexity in adult mice is a function of terminal end bud activity during juvenile ductal extension. Morphometric analysis showed that the branching complexity of 11-week-old *Lrp6^+/−^* glands was decreased by 17% compared with that of littermate wild-type mice (*p* = 8.4×10^−3^) (Fig. 3C). We measured the weight of *Lrp6^+/−^* and *Lrp6^+/+^* mammary fat pads but no difference was detected (data not shown).

We next tested whether the *Lrp6^+/−^* ductal phenotype would become more or less apparent after transplantation of epithelial cells into wild-type mammary glands. For this purpose we transplanted 5,000 *Lrp6^+/−^* or *Lrp6^+/+^* mammary epithelial cells into the cleared fat pads of 21-day-old immune-compromised *Rag2*-deficient females. Out-growths were found in 5/8 and 6/8 fat pads transplanted with *Lrp6^+/−^* and *Lrp6^+/+^* mammary epithelial cells, respectively. The branching morphology was similar in host fat pads six weeks after surgery regardless of transplant genotype (Fig. 3D). None of the 4 *Rag2*-deficient control mice whose fat pads (*n* = 8) were cleared but not injected with mammary epithelial cells contained ductal out-growths, confirming our ability to successfully clear the mammary fat pad (Fig. 3D).

We also examined the effect of compound mutations of *Lrp6* and *Lrp5*. While embryos of the *Lrp6^−/−^;Lrp5^−/−^* and *Lrp6^−/−^;Lrp5^+/−^* genotypes die during embryogenesis, some *Lrp6^+/−^;Lrp5^−/−^* mice live into adulthood. *Lrp6^+/−^;Lrp5^−/−^* mice have limb deformities and 80% die shortly after birth, but surviving pups have a normal life span and are fertile [16], [17]. We dissected the mammary glands from formalin-fixed *Lrp6^+/−^;Lrp5^−/−^* female carcasses previously studied for their bone phenotype. Although nipples and normal size fat pads were visible upon dissection, none of the 5 analyzed animals had ductal tree outgrowths in any of their 10 mammary glands (Fig. 3E).

### *MMTV-Wnt1* expression induces epithelial proliferation in *Lrp6^−/−^* and *Lrp6^+/−^* mice

**Figure 4:**
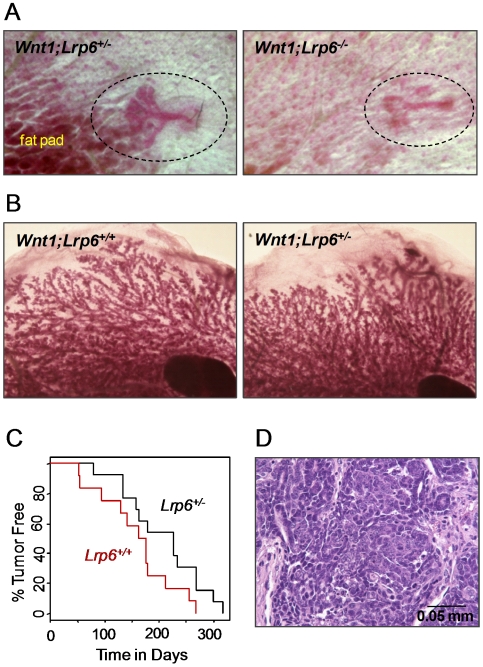
Wnt1-induced mammary tumorigenesis in *Lrp6^+/−^* females. Shown in (A-B) are representative carmine stained skin pads and mammary whole mounts. (A) Skin pads collected from E18.5 *MMTV-Wnt1;Lrp6^+/+^* and *MMTV-Wnt1;Lrp6^−/−^* embryos. (B) Mammary whole mounts collected from adult *MMTV-Wnt1;Lrp6^+/+^* and *MMTV-Wnt1;Lrp6^+/−^* females. (C) The percentages of *MMTV-Wnt1;Lrp6^+/+^* (*n* = 12) and *MMTV-Wnt1;Lrp6^+/−^* mice (*n* = 13) that were tumor-free (as determined by weekly visual inspection and/or palpation) were plotted against the age when tumors were found. (D) Standard histopathological evaluation showed that all *Lrp6^+/+^* and *Lrp6^+/−^ MMTV-Wnt1* tumors are moderately differentiated alveolar mammary adenocarcinomas.

Female mice that express Wnt1 under the mammary-specific MMTV promoter reproducibly develop mammary adenocarcinomas within one year [26]. The tumors develop in a context of widespread hyperplasia that is noticeable as early as E18.5. To test whether transgenic expression of Wnt1 could induce ductal side-branching in *Lrp6^−/−^* embryos and to determine the requirement for *Lrp6* in Wnt1-induced tumorigenesis, we intercrossed *Lrp6^+/−^* and *MMTV-Wnt1* mice. We found that transgenic expression of Wnt1 partially rescued the *Lrp6^−/−^*ductal phenotype at E18.5 and induced sprout elongation and side-branching (Fig. 4A). The inguinal mammary epithelial tree of *MMTV-Wnt1;Lrp6^+/+^* (*n* = 6) and *MMTV-Wnt1;Lrp6^−/−^* (*n* = 4) embryos contained on average 10 and 4 end buds, respectively. The *MMTV-Wnt1;Lrp6^−/−^* mammary nipples and fat pads were still abnormally small. Furthermore, no obvious reduction in mammary hyperplasia was observed, and tumor onset was only slightly delayed in adult *MMTV-Wnt1;Lrp6^+/−^* females (*p* = 0.08) (Fig. 4B–C). Histopathological examination showed that all tumors, regardless of *Lrp6* genotype, were moderately differentiated alveolar mammary adenocarcinomas (Fig. 4D).

### Increased *Lrp6* expression in human basal-like breast cancer

**Figure 5:**
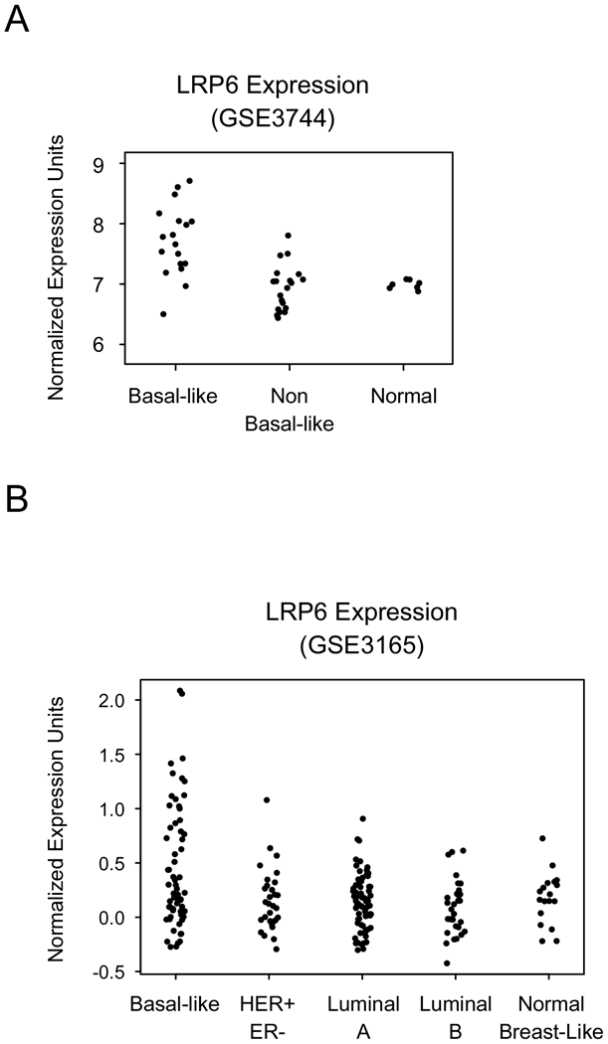
Increased *Lrp6* expression in basal-like human breast cancer. The relative expression of *Lrp6* in breast cancer samples analyzed by Affymetrix and organized into defined subgroups. Each dot represents the relative expression of *Lrp6* in one tumor sample. (A) Expression data was obtained from [30]. *Normal* includes samples obtained from normal breast tissue. (B) Expression data was obtained from [31]. *Normal Breast-like* includes samples obtained from normal and cancerous breast tissue that exhibited expression profiles similar to that of normal breast tissue. A subset of breast cancers within the basal-like subgroup of both studies exhibit increased expression of *Lrp6*.

Global gene expression analyses of human breast cancers have identified four major tumor subtypes and a normal breast tissue group [27]–[29]. Two subtypes are estrogen receptor (ER)–negative and have poor patient outcomes: one of these two subtypes is defined by the high expression of HER2, and the other shows characteristics of basal/myoepithelial cells (basal-like). The remaining two subtypes, luminal subtype A or B, are ER-positive and Keratin 8/18–positive. We looked at the expression pattern of *Lrp6* in two published transcriptome profiles of breast cancer. The first study, led by Livingston and Ganesan at Harvard Medical School, contained 45 samples (18 Basal-like, 20 non-Basal, and 7 Normal) [30] (Fig. 5A) and the second, led by Perou at the University of North Carolina, contained 213 samples (64 Basal-like, 30 HER+, 70 Luminal A, 31 Luminal B, and 18 Normal Breast-like) [31] (Fig. 5B). We found that increased expression of *Lrp6* was associated with basal-like breast cancer (*p* = 1.8×10^–4^, *p* = 9.6×10^–5^) (Fig. 5A–B). In fact, a fraction of samples within the basal-like subgroup of both studies expressed levels of *Lrp6* not seen in any of the other subgroups or the normal controls. Interestingly, we also found a correlation between *Lrp5* expression and basal-like breast cancer (*p* = 4.6×10^–3^, *p* = 3.7×10^–3^) (data not shown).

## Discussion

Wnt signaling pathways play essential roles at multiple steps of animal development, including embryonic induction, organogenesis, and adult tissue homeostasis. The binding of Wnt ligands to a receptor complex composed of one Frizzled (Fzd) protein and either LRP5 or LRP6 initiates the Wnt/β-catenin cascade. At the cellular level, Wnt/β-catenin signaling regulates a broad range of functions, including the self-renewal and differentiation of stem cells. We have previously shown that Lrp5 is critical for mammary ductal stem cell activity: its loss is associated with impaired mammary gland development and delayed Wnt1-induced mammary tumorigenesis [21]. Here, we show that Lrp6 is also required for normal mammary gland development. Loss of *Lrp6* impairs embryonic mammary development, evident by abnormal mammary placode development at E12.5 and the absence of ductal branching and fat pad development at E18.5 (Fig. 2A–D). Furthermore, postnatal mammary development is affected in *Lrp6* heterozygote females: the number of TEBs and ductal branches are reduced in juvenile and adult mice, respectively (Fig. 3A–C). Although it is not yet clear whether *Lrp6* is required for ductal stem cell activity, we found that *Lrp6* is expressed by epithelial cells within the basal cell layer that also express stem cell markers (Fig. 1B–D). Finally, an increased expression of *Lrp6* is associated with human basal-like breast cancer (Fig. 5A–B). Taken together with our previous study, our results show that both Lrp5 and Lrp6 play essential roles in the mammary gland development and cannot fully compensate for each other's loss.

The initial stages of mammary development are hormone-independent, depending instead on reciprocal signaling between the epithelium and the mesenchyme (reviewed in [32]–[34]). Mammary gland development begins at about embryonic day 10.5 with the appearance of the mammary lines. In response to signals from the underlying mesenchyme, the mammary lines give rise to five pairs of lens-shaped mammary placodes that subsequently invaginate into the underlying dermal mesenchyme, forming mammary buds. In females at E15.5, the buds elongate and form a mammary sprout that extends towards the fat pad precursor mesenchyme. Mammary nipples are formed around E16.5. Meanwhile the epithelial sprout branches into the fat pad precursor mesenchyme, resulting in the formation of a rudimentary ductal tree prepared to respond to hormonal cues at puberty.

Studies using Wnt reporter gene mouse strains have shown that activation of the Wnt/β-catenin signaling pathway along the mammary lines coincides with the initiation of mammary morphogenesis and subsequently localizes to the mammary placodes, buds, and rudimentary ductal tree [21], [35], [36]. Furthermore, Wnt signaling appears to be required for embryonic mammary development: embryos deficient for *Lef1* fail to develop/maintain their mammary placodes, and embryos that express the Wnt inhibitor Dkk1 in developing epithelium fail to form mammary placodes [37], [38]. Dkk1 inhibits the Wnt signaling pathway by binding to (and presumably inactivating) Lrp5 and Lrp6 [39]. In agreement, embryos lacking *Lrp5* exhibit reduced Wnt/β-catenin signaling and have significantly smaller mammary placodes than littermate wild-type controls [21]. Despite the placode phenotype, the rudimentary ductal tree and fat pad develop normally in *Lrp5^−/−^* embryos.

At E12.5, the phenotype of *Lrp6^−/−^* embryos is similar to that of *Lrp5^−/−^* embryos, the mammary placodes are smaller, abnormally developed, and contain few cells with activated Wnt/β-catenin signaling relative to wild-type controls (Fig. 2C–D). However, in contrast to *Lrp5^−/−^* embryos, the rudimentary ductal tree fails to develop in *Lrp6^−/−^*embryos (Fig. 2A). It is not clear if this phenotype is due to an inadequacy of the mammary epithelium or/and the fat pad. Signals from the fat pad mesenchyme play a central role for ductal elongation and branching and may be diminished in the absence of *Lrp6* [32]–[34]. *Lrp6* is normally expressed both in the mammary epithelium and fat pad, and the loss of *Lrp6* is associated with abnormal development of both compartments. The *MMTV-Wnt1;Lrp6* cross showed that *Lrp6^−/−^* mammary epithelium is not incapable of further development, since transgenic expression of *MMTV-Wnt1* produced sprout elongation and branching despite the presence of underdeveloped fat pads (Fig. 4A). This suggests that the dose of Wnt/β-catenin signaling within the mammary epithelium is critical and can determine the degree of embryonic ductal development. In further support of this, *Lrp6^+/−^;Lrp5^−/−^* female mice fail to develop mammary ducts (Fig. 3E).

We have previously shown that *Lrp5* is required for Wnt1-induced mammary tumorigenesis [21]. Tumor onset is dramatically delayed in *MMTV-Wnt1;Lrp5^−/−^* and *MMTV-Wnt1;Lrp5^+/−^* females. The effect of *Lrp6* heterozygosity was much less pronounced. The average time of tumor onset was delayed by 6 and 17 weeks in *Lrp6^+/−^* and *Lrp5^+/−^* mice, respectively (Fig. 4C and [21]). *Lrp6* heterozygosity slightly delayed the time at which tumors first appeared, but the rate of tumor onset was then similar between *Lrp6^+/−^* and *Lrp6^+/+^* females (Fig. 4C). Consistent with this, we saw no reduction in mammary hyperplasia in adult *MMTV-Wnt1;Lrp6^+/−^* females (Fig. 4B). Relatively little is known of the ligand-receptor specificity exhibited by different Wnts, Fzds, and LRPs in Wnt signaling. However, our results suggest that Lrp5 plays a pivotal role over Lrp6 in transmitting oncogenic Wnt signals in the *MMTV-Wnt1* mammary tumor model.

Wnt/β-catenin signaling is a key regulator of embryonic and somatic stem cells [9], [10]. In the adult mouse, Wnt/β-catenin signaling has been shown to regulate a number of epithelial stem cell compartments, including those of the skin, gut, and mammary gland [40]–[44]. Mammary epithelial cells with stem cell activity, i.e., cells that can develop a functional ductal tree in single cell transplantation experiments, can be identified by FACS [23], [45]. These cells typically express moderate and high levels of the cell surface markers CD24 and CD49f, respectively [23]. Interestingly, most *Lrp6*-expressing mammary epithelial cells exhibited this expression pattern (Fig. 1C–D). Furthermore, mammary stem cells are presumed to reside within the basal cell layer of the mature mammary ducts [23], [46], [47], and this is where *Lrp6*-expressing cells primarily were identified (Fig. 1B). We also found that human basal-like breast cancers are associated with increased *Lrp6* expression, suggesting that these tumors may be enriched with *Lrp6*-expressing cells. Subpopulations of cancer cells with stem cell properties are especially frequent within basal-like breast cell lines and show increased tumorigenic and invasive potential [48], [49]. Whether Lrp6 is required for ductal stem cell activity remains to be determined, as does the role of Lrp6-mediated Wnt signaling in the mammary fat pad. Development of conditional mouse models for *Lrp6* and *Lrp5* deletion will aid in the understanding of the specific roles of these proteins in mammary gland development and tumorigenesis. This is particularly important because Wnt/β-catenin signaling is often activated in human breast cancer. At least 50% of human breast cancers exhibit nuclear/cytoplasmic β-catenin, and aberrant activation of the pathway at the receptor level is common [9], [50]–[52]. Hence, therapeutic interventions targeting LRP5 and/or LRP6 could be useful in treating some types of breast cancer, particularly the basal-like class for which few, if any, effective treatments exist.

## Materials and Methods

### Mouse strains and husbandry

*Lrp6* (C57Bl/6J) knock-out mice, as well as *BAT-gal* (FVB/N) and *MMTV-Wnt1* (FVB/N) transgenic mice, have been previously described [13], [25], [26]. PCR-based strategies were used to genotype these mice (details available upon request). All experiments performed were approved in advance by the Van Andel Research Institute Institutional Animal Care and Use Committee. To assay the appearance of mammary tumors, the mice were inspected three times a week and were euthanized when tumors appeared.

### Mammary gland morphogenesis

Whole mounts were prepared as described [53]. Briefly, inguinal mammary glands were dissected, fixed overnight in Carnoy formula (6∶3∶1 ratio of ethanol:chloroform:glacial acetic acid), rehydrated, and stained overnight in Carmine alum stain. The stained glands were dehydrated, cleared in xylene, and stored in Methyl Salicylate. After whole-mount pictures had been taken, the tissues were embedded in paraffin for sectioning. Sections (5 µm) were rehydrated and counterstained with H&E.

### Oil red O staining

Ventral skin pads were placed in propylene glycol for 2 min and then in Oil red O stain heated to 60°C for 6 min, followed by 85% propylene glycol in distilled water for 1 min, and lastly rinsed twice in water.

### X-gal staining

*Lrp6^+/−^* and *Lrp6* ^+/+^ (negative control) mammary glands and 12.5-day-old embryos were isolated and fixed in 0.25% glutaraldehyde, 2% formaldehyde, 5 mM EGTA, and 2 mM MgCl_2_ in PBS pH 7.4 at 4°C. Mammary glands were fixed for 2 h; embryos for 1 h. Glands and embryos were then rinsed twice in 2 mM MgCl_2_, 0.1% sodium deoxycholate, and 0.2% NP40 in PBS at room temperature for 1 h and then were stained in X-gal buffer (1 mg/ml X-gal, 2 mM MgCl_2_, 0.01% sodium deoxycholate, 0.02% NP-40, 5 mM Fe_3_(CN)_6_, 5 mM Fe_4_(CN)_6_ in PBS) at 30°C. *Lrp6^+/−^* mammary glands were stained overnight; *BATgal* transgenic embryos were stained for 20 min. The whole mounts were then rinsed in PBS, dehydrated, and cleared in xylene. After whole-mount pictures had been taken, the tissues were immediately embedded in paraffin for sectioning. Sections (5 µm) were counterstained with 0.1% nuclear fast red or eosin. A minimum of three animals per genotype and time point were analyzed.

### Primary mammary epithelial cell isolation

Mammary glands were dissected and minced with scissors, then the cells were dissociated for 8 h at 37°C in EpiCult-B with 5% fetal bovine serum, 300 units/ml collagenase, and 100 units/ml hyaluronidase. After vortexing and lysis of the red blood cells in NH_4_Cl, a single-cell suspension was obtained by sequential dissociation of the fragments by gentle pipetting for 1–2 min in 0.25% trypsin and then for 2 min in 5 mg/ml Dispase II plus 0.1 mg/ml DNase I, followed by filtration through a 40-mm mesh. All reagents were from StemCell Technologies Inc.

### Mammary transplantation assays

Viable mammary epithelial cells collected from 3-month-old *Lrp6^+/−^* (*n* = 5) and *Lrp6^+/+^* (*n* = 3) virgin female mice were counted on a hemocytometer, suspended at the desired concentration in 1∶1 PBS:Matrigel (BD Biosciences) together with 0.5% trypan blue loading dye, and kept on ice until transplantation. Cells were injected in a total volume of 10 µl into contralateral cleared fat pads of the #4 mammary glands of 21-day-old female *Rag2^−/−^* mice using a Hamilton syringe [54]. Six or 12 weeks after transplantation, the fat pads were dissected, processed, and stained with carmine as described above.

### Fluorescence-activated cell sorting

Mammary epithelial cells were isolated as described above. The Mouse Mammary Stem Cell Enrichment Kit from StemCell Technologies was used to obtain CD45, Ter119, and CD31 triple-negative cell suspensions labeled with CD24 and CD49f. Briefly, mammary epithelial cells were first incubated in a cocktail of biotinylated CD45, Ter119, and CD31 antibodies, and then were exposed to a biotin selection cocktail and removed using magnetic nanoparticles. The remaining cells were labeled with DDAOG (10 µm), CD24-PE, and CD49f-FITC for 30 min on ice, then washed and resuspended in Hanks with 2% FBS and kept on ice. Live cells were discriminated by propidium iodine exclusion. Cell sorting and analysis was done by using the BD FACSCalibur Flow Cytometer and Cell quest 5.2.1 software, respectively (BD Biosciences). The FACS analysis described above was repeated twice and included mammary epithelial cells collected from 4 *Lrp6^+/−^* and 5 *Lrp6^+/+^* females.

### Expression profiling

Processed expression chip used in the GSE3744 dataset contained multiple probes that mapped to LRP6. Therefore in this dataset, the average *LRP6* expression value was computed for each sample and used in subsequent analysis. In the GSE3165 dataset, three related expression chips were used and a single *LRP6* probe (NM_002336) was present across the majority of the chips. Therefore, the expression value derived from the NM_00236 probe was used in subsequent analysis. Within each dataset, the *LRP6* expression values were partitioned into groups based on tumor subtype and differences in expression between the basal-like and non-basal like samples evaluated using a two-sided Welsh's t-test.
